# Elevated Phospholipase A_2_ Activities in Plasma Samples from Multiple Cancers

**DOI:** 10.1371/journal.pone.0057081

**Published:** 2013-02-22

**Authors:** Hui Cai, Elena G. Chiorean, Michael V. Chiorean, Douglas K. Rex, Bruce W. Robb, Noah M. Hahn, Ziyue Liu, Patrick J. Loehrer, Marietta L. Harrison, Yan Xu

**Affiliations:** 1 Department of Obstetrics and Gynecology, Indiana University School of Medicine, Indianapolis, Indiana, United States of America; 2 Department of Thoracic Oncosurgery, First Affiliated Hospital of Xi'an Jiaotong University, Xi'an, People's Republic of China; 3 Department of Medicine, Indiana University Melvin and Bren Simon Cancer Center, Indiana University School of Medicine, Indianapolis, Indiana, United States of America; 4 Hoosier Oncology Group, Indianapolis, Indiana, United States of America; 5 Department of Biostatistics, Indiana University School of Medicine, Indianapolis, Indiana, United States of America; 6 Medicinal Chemistry and Molecular Pharmacology, Oncological Sciences Center, Purdue University Center for Cancer Research, West Lafayette, Indiana, United States of America; Johns Hopkins University, United States of America

## Abstract

Only in recent years have phospholipase A_2_ enzymes (PLA_2_s) emerged as cancer targets. In this work, we report the first detection of elevated PLA_2_ activities in plasma from patients with colorectal, lung, pancreatic, and bladder cancers as compared to healthy controls. Independent sets of clinical plasma samples were obtained from two different sites. The first set was from patients with colorectal cancer (CRC; n = 38) and healthy controls (n = 77). The second set was from patients with lung (n = 95), bladder (n = 31), or pancreatic cancers (n = 38), and healthy controls (n = 79). PLA_2_ activities were analyzed by a validated quantitative fluorescent assay method and subtype PLA_2_ activities were defined in the presence of selective inhibitors. The natural PLA_2_ activity, as well as each subtype of PLA_2_ activity was elevated in each cancer group as compared to healthy controls. PLA_2_ activities were increased in late stage vs. early stage cases in CRC. PLA_2_ activities were not influenced by sex, smoking, alcohol consumption, or body-mass index (BMI). Samples from the two independent sites confirmed the results. Plasma PLA_2_ activities had approximately 70% specificity and sensitivity to detect cancer. The marker and targeting values of PLA_2_ activity have been suggested.

## Introduction

Cancer is one of the major health burdens worldwide. More than 1.6 million new cancer cases and over 577,000 deaths from cancer are projected to occur in the United States in 2012 [1]. Lung cancer (LC) and colorectal cancer (CRC) are among the most frequent types of cancers. Pancreatic and bladder cancers count for (3–7% of all cancers), but pancreatic cancer (PC) is one of the deadest cancers, with a dismal 5-year survival rate of 6% [1]. The success of current cancer treatments depends strongly on the time of diagnosis, with early detection and identification of high risk patient populations resulting in the most favorable overall survivals in these diseases [1], [2]. It is expected that early detection and screening of high risk patient populations may have the most significant impact on altering overall survival in these diseases. Thus, there is an urgent need for efficient and sensitive early detection methods [3]. While imaging-based detection methods, including spiral computerized axial tomography (CT) and colonoscopy are effective early detection methods for LC and CRC, respectively [4], they are rather expensive and inconvenient. In addition, modern imaging techniques have a high incidence of discovery of lung nodules, many of which are falsely positive, but still calling for additional and sometimes painful examinations [5]. Molecular approaches to cancer diagnosis through biomarkers measured by non-invasive means could significantly improve the specificity and sensitivity of cancer detection [5]. In particular, serologic biomarkers may be the most useful in early detection due to the minimal invasiveness, cost-effectiveness, and convenience. Moreover, serological biomarkers identifying novel cancer-related genes and/or molecules may provide new insight into the biology of cancers and may also become attractive targets for treatment [3].

Phospholipase A_2_ enzymes (PLA_2_s) are the major enzymes producing the cyclooxygenase-2 (COX-2) substrate, arachidonic acid (AA), as well as lysophospholipids. Both of these classes of products are signaling molecules involved in cancers. However, only in recent years have PLA_2_s emerged as cancer targets [6]. More than 30 enzymes that possess PLA_2_ or related activity have been identified in mammals [7]. They are divided into four groups based on their cellular localization, substrate specificity, and calcium-dependence [8], including cytosolic (cPLA_2_), calcium-independent (iPLA_2_), secreted (sPLA_2_), and lipoprotein-associated PLA_2_ (Lp-PLA_2_). In cancer, most of the attention has been focused on sPLA_2_ and cPLA_2_ [8]. We and others have shown that iPLA_2_ is functionally involved in promoting the development of ovarian cancer (OC) and other cancers, *in vitro* and *in vivo* [9]–[13]. sPLA_2_ and Lp-PLA_2_ are secreted enzymes. In contrast, both cPLA_2_ and iPLA_2_ are cytosolic enzymes and their extracellular existence has only been shown to be related to exosomes from RBL-2H3 cells (a mast and basophil cell line) [14]. Exosomes are 40–100 nm diameter membrane vesicles released from multivesicular bodies by intact cells and are known to participate in intercellular signaling [15]. We have recently detected extracellular- and exosome-free iPLA_2_ and cPLA_2_ activities in ascites and tissues from OC patients [16].

In the current work we have focused on PLA_2_ activities rather than expression of individual PLA_2_ enzymes and examined PLA_2_ activities in blood plasma samples from patients with different cancers, in comparison with those from healthy controls. We used our recently validated quantitative, convenient, highly reproducible PLA_2_ assay method [16] and showed for the first time that plasma PLA_2_ activities from patients with CRC, LC, PC, and bladder cancer (BC) were significantly higher than those of healthy controls. In addition, PLA_2_ activities were correlated with tumor stages in CRC. Other potential influential factors, including sex, age, smoking and alcohol consumption were also examined in this study.

## Materials and Methods

### Human sample collection and processing

Two sets of independently collected clinical plasma samples were obtained for this study. The first set from the Indiana University School of Medicine (IUSM) focused on CRC patients and healthy controls screened by colonoscopy and found negative for adenomatous polyps and CRC. Blood samples collected in the presence of EDTA were centrifuged at 1,750 g for 15 min at 15–24°C, aliquoted in siliconized Eppendorf tubes and stored at −80°C. The second set of samples was from patients with lung, bladder, or pancreatic cancers, as well as healthy controls. These samples were collected by the Hoosier Oncology Group (HOG) in Indianapolis, IN as part of the study entitled, “A Biological Sample Collection Protocol of Patients with and without Metastatic Solid Organ Malignancies: Hoosier Oncology Group Study BANK09-138”. Blood samples were centrifuged at 3,500 rpm for 30 min. The aliquoted samples were stored at −70°C. HOG is a non-profit medical research organization. Although it is a separate entity, IUSM is a supporting organization for board appointments and IRB approvals for HOG. Three separate IRB protocols for collecting and/or use the blood samples related to this work have been approved by the same IUSM Institutional Review Board (Protocol numbers: 0670-81, 0808-24, 0905-20). Written informed consent forms were obtained from all subjects and all clinical investigation had been conducted according to the principles expressed in the Declaration of Helsinki.

### Reagents and inhibitors

The PLA_2_ substrate 1-O-(6-Dabcyl-Aminohexanoyl)-2-O-(6-(12-BODIPY-Dodecanoyl) Aminohexanoyl)-sn-3-Glyceryl Phosphatidylcholine (DBPC) was from Echelon Bioscience (Salt Lake City, UT, USA). Bromoenol lactone (BEL) and methyl arachidonylfluorophosphonate (MAFP) were from Santa Cruz Biotechnology (Santa Cruz, CA, USA).

### PLA_2_ enzymatic activity analyses

PLA_2_ activities were analyzed using the fluorescent substrate DBPC, a fluorogenic phosphatidylcholine substrate [17]. Plasma samples (0.1 µL) were mixed with DBPC (0.2 µg in PBS) to final volume 200 µL. The fluorescence was read at intervals over several hours on a Victor^3^V plate reader (Perkin Elmer, Waltham, MA, USA). PLA_2_ activities were expressed as change in fluorescence intensity/min/µL of plasma. As previously described [16], we selected conditions to distinguish PLA_2_ activity derived from different subtypes: a) the “natural” PLA_2_ activity without any exogenous additives, b) the iPLA_2_ activity in the presence of 5 mM EDTA (a divalent cation chelator to block all PLA_2_s requiring calcium, including sPLA_2_ and cPLA_2_), c) the sPLA_2_ activity in the presence of 1.2 mM calcium chloride (the natural ionized calcium concentration in blood [18]) and MAFP (10 µM, a dual inhibitor of cPLA_2_ and iPLA_2_), and d) the cPLA_2_ activity in the presence of 100 µM calcium chloride and bromoenol lactone (BEL, 10 µM, a selective inhibitor for iPLA_2_).

### Statistical Analysis

Categorical variables were summarized as counts with percentages and continuous variables were summarized as means with standard deviations (SD) across the healthy control, and cancer groups. The Chi-square test was used to test the associations between disease statuses and categorical covariates such as sex, race, smoking, and alcohol drinking. For continuous covariates such as age and BMI, one-way ANOVA was used to test the overall difference and Student *t*-test was used to test the pair-wise difference across disease statuses. Linear regression was used to assess the univariate association between PLA_2_ measurements and covariates. Logistic regression was used to evaluate the classification performance of PLA_2_ measurements. Bootstrap was used to internally validate the classification performance using 1000 samples [19]. Each sample had the same size as the original data set and was used to develop the model. The results were then tested in the original data. Areas under the curve (AUCs) were calculated for each repeat. All tests were two-sided. Given the exploratory nature of this study, no adjustments for multiple comparisons were adopted. *P* values <0.05 were considered to be statistically significant. All analyses were performed using SAS software Version 9.3 (SAS Institute Inc., Cary, NC, USA).

## Results

### Subject demographic data

The demographic data for the study participants are summarized in **Table 1**. For the IUSM CRC set samples, 41.7% of the overall participants were male, 88.7% were white, and the mean age was 53.1 years. The age, sex, and race composition were not significantly different in control vs. CRC groups (*P* values >0.05; **Table 1**). For the second set of samples, the age in the healthy control group was significantly younger than each of the three cancer groups (**Table 1**). Although males were dominant in each group, they were not significantly different from the second set of healthy controls. 71.2% of the overall participants were male, 90.4% were white, and the mean age was 58.3 years (**Table 1**).

**Table 1:** Demographic data for participants from IUSM and HOG study.

		Age (years)			Sex (%)			Race (%)			
	No.	Mean	SD	*P* value	Male	Female	*P* value	White	African American	Others	*P* value
Participants from IUSM											
Healthy	77	51.6	13.5		40.3	59.7		89.6	9.1	1.3	
CRC	38	56.0	13.4	0.1075	44.7	55.3	0.6470	86.8	5.3	7.9	0.4501
Participants from HOG											
Healthy	79	42.7	12.0		77.2	22.8		89.3	2.7	8.0	
LC	95	63.2	9.5	<0.0001	64.2	35.8	0.0621	89.5	8.4	2.1	0.0654
BC	31	70.5	10.0	<0.0001	87.1	12.9	0.2438	93.6	6.5	0.0	0.1868
PC	38	68.7	8.7	<0.0001	63.2	36.8	0.1102	92.1	5.3	2.6	0.4327

CRC: colorectal cancer; LC: Lung cancer; BC: Bladder cancer; PC: Pancreatic cancer; SD, standard deviation; HOG: Hoosier Oncology Group.

In the HOG set: • For gender, the overall *P* value is 0.0339. • For Age, the overall *P* value is <0.0001. • For race, the overall *P* value is 0.2352. Since it was not significant, it was not recommended to perform pair-wise comparisons. Nonetheless, *P* values for pair-wise comparisons are given to be consistent with the other two variables. All pair-wise comparisons are against healthy controls.

### Reproducibility and stability of the PLA_2_ assays

We have recently validated the quantitative nature of the DBPC-based PLA_2_ assays and optimized the conditions for biological fluids and tissues samples [16]. When each of 8 representative plasma samples were analyzed three times, the PLA_2_ activity values reproducible, with an average standard deviation (SD) 13.5 (1.3%). We also tested the PLA_2_ activity stability by comparing them in samples before storage to those in the same samples stored at −80°C for one month, as well as the effect of freeze-and-thaw on the PLA_2_ activities in 8 representative samples. As previously reported [16], PLA_2_ activities were stable with SDs <35.8 (5.8%). In addition, we showed that in the presence of all other assay components including inhibitor(s) used in this study, but in the absence of blood or other biological samples, the non-specific hydrolysis of the DBPC substrate was negative and negligible (data not shown).

### The natural PLA_2_, cPLA_2_, iPLA_2_, and sPLA_2_ activities were elevated in cancer samples vs. healthy controls

Blood iPLA_2_ and cPLA_2_ activities have not been previously reported in human samples. We analyzed the natural PLA_2_ (defined as the PLA_2_ activities measured under the conditions without any modifiers, which may be lower than the sum of the subgroup PLA_2_ activities measured under modified conditions), as well as cPLA_2_, iPLA_2_, and sPLA_2_ activities in plasma samples.

**Figure 1:**
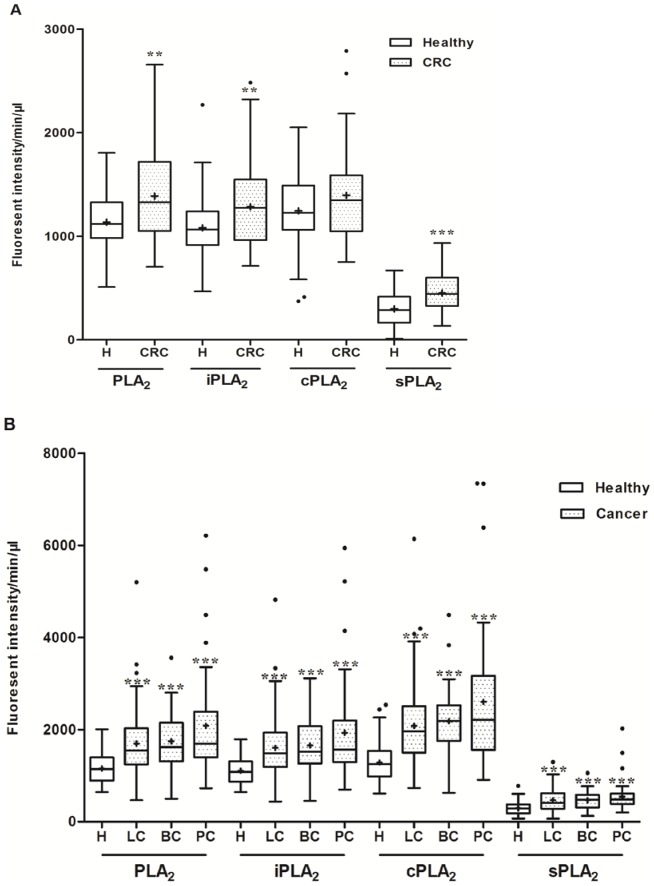
Plasma PLA_2_ activities were elevated in cancer groups vs. healthy controls. Comparison of PLA_2_ activities in the healthy control (normal colonoscopy; n = 77) and CRC (n = 38) groups. A. Comparison of PLA_2_ activities in the healthy (n = 79) and other cancer groups. Lung cancer (LC, n = 95), bladder cancer (BC, n = 31) and pancreatic cancer (PC, n = 38). The distribution of the natural and individual groups of PLA_2_ activities were analyzed as described in Materials and Methods. The mean values of PLA_2_ activities were presented by the “+” in the figure. * *P*<0.05; ** *P*<0.01; *** *P*<0.001.

Except for the healthy vs. CRC cPLA_2_ activity, all other comparisons showed that PLA_2_ activities were significantly elevated in the cancer groups (**Fig. 1A** and **1B**). PLA_2_ activity values across plasma samples from LC, BC, and PC patients were not significantly different (*P* values >0.05).

Since sPLA_2_ is secreted, it was expected that sPLA_2_ activity would account for the major portion of the PLA_2_ activity detected in the blood. However, in our results, high levels of iPLA_2_ and cPLA_2_ and low levels of sPLA_2_ activities were observed in all plasma samples. Blood exosomes may be the carriers of these activities [15]. We used differential centrifugation steps as we described recently [16] to determine whether these activities could be associated with platelets, exosomes, or other microvesicles. Two blood samples were first centrifuged at 1,750 g for 15 min to remove most blood cells and the supernatant was termed S1. S1 was centrifuged at 20,000 *g* for 20 min, which resulted in S2 and the pellet 2 (P2; cell fragments and large vesicles). S2 was ultracentrifuged at 110,000 *g* for 2 hr, which resulted in S3 and P3 (exosomes). A final centrifugation of S3 at 200,000 *g* for 2 hr resulted in S4 and P4 (other microvesicles). We found that all PLA_2_ activities were retained in the S1 through S4 fractions, suggesting they were “free” and not associated with microvesicles (detailed data not shown). This is similar to what we have recently reported in human ovarian cancer ascites and novel secretion mechanisms for iPLA_2_s and cPLA_2_s may be involved [16].

### Clinical parameters and PLA_2_ activities in cancer plasma samples

**Figure 2:**
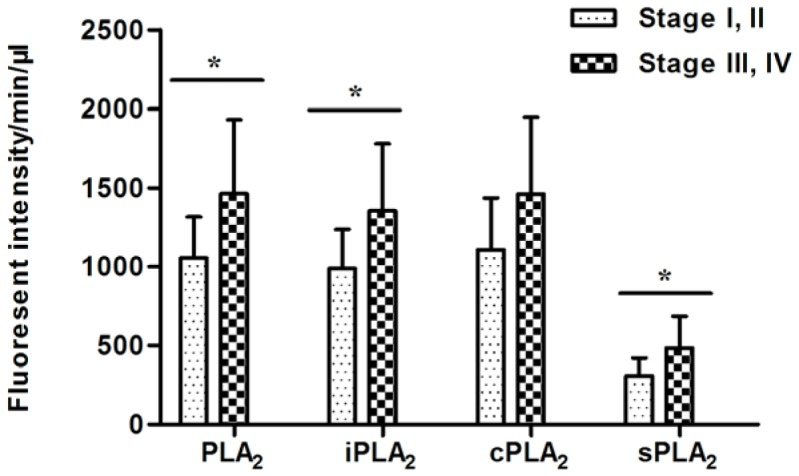
PLA_2_ activities were higher in late stage CRC samples. The natural and individual groups of PLA_2_ activities were progressively increased by tumor stage. Stage I and II (n = 7), Stage III and IV (n = 31). *Student t-test, *P*<0.05.

Interestingly, the natural, iPLA_2_, and sPLA_2_ activities in plasma of CRC patients were increased in subjects with late stages (III and IV) as compared to earlier stage (I and II) disease (*P* = 0.0335, 0.0367, 0.0778, and 0.0345 for PLA_2_, iPLA_2_, cPLA_2_, and sPLA_2_, respectively; **Fig. 2**), suggesting that these enzymatic activities may be involved in CRC progression. We compared the PLA_2_ activities in colon (n = 24) vs. rectal (n = 14) samples. Even though the nature, as well the sub-group PLA_2_ activities in colon cancer patients were higher than those from rectal cancer patients, the differences were not statistically significant (*P*>0.05 in all cases; **Table 2**). In addition, no difference was found in PLA_2_ activities among different subgroups of CRC patients with previous treatments (surgery, chemotherapy, radiotherapy, or untreated; **Table 2**). In contrast, we found that PLA_2_ activities were not correlated to either T or N stages of LC, BC, or PC (**Table 3**).

**Table 2:** Clinical characteristics and PLA_2_ activities in the CRC cases.

		PLA_2_			iPLA_2_			cPLA_2_			sPLA_2_		
	No.	Mean	SD	*P* value	Mean	SD	*P* value	Mean	SD	*P* value	Mean	SD	*P* value
Primary site				0.6856*			0.7407*			0.6419*			0.3410*
Colon	24	1412.3	473.75		1302.8	428.78		1424.0	480.16		476.3	199.33	
Rectum	14	1348.0	460.33		1254.7	428.33		1347.7	489.95		408.3	195.50	
Previous treatment				0.6543**			0.3081**			0.3348**			0.6212**
Surgery only	7	1340.3	382.29		1193.1	286.55		1303.4	424.30		435.5	92.75	
CT and RT	3	1614.8	542.80		1599.5	626.64		1755.4	707.50		495.9	219.34	
Surgery and CT	10	1496.8	596.54		1399.5	543.97		1521.1	633.55		519.8	264.44	
Surgery, CT and RT	5	1459.0	437.46		1402.9	322.07		1511.8	322.27		472.8	200.95	
Untreated	13	1252.2	404.71		1128.6	341.99		1221.8	327.50		388.5	183.68	

* Student *t*-test.

** One-way ANOVA.

CT: chemotherapy; RT: radiotherapy.

**Table 3:** Clinical characteristics and PLA_2_ activities in the LC, BC, and PC cases.

		PLA_2_			iPLA_2_			cPLA_2_			sPLA_2_		
	No.	Mean	SD	*P* value	Mean	SD	*P* value	Mean	SD	*P* value	Mean	SD	*P* value
Lung cancer													
T stage				0.4760**			0.5001**			0.4960**			0.6129**
T1	19	1714.2	548.5		1626.6	514.2		2190.2	752.6		457.1	280.7	
T2	21	1569.3	671.8		1490.6	640.6		1924.1	754.0		478.7	261.4	
T3	9	1437.9	479.0		1347.4	460.7		1721.3	480.5		356.2	166.6	
T4	35	1803.5	878.2		1691.8	823.9		2141.7	1105.9		471.5	228.4	
N stage				0.9973**			0.9685**			0.9758**			0.8009**
N0	14	1626.7	566.2		1559.5	522.6		2061.3	584.4		386.3	143.2	
N1	11	1574.8	508.1		1442.7	479.8		1960.3	671.2		404.1	200.4	
N2	21	1613.9	643.7		1524.1	631.4		1961.6	883.6		434.1	222.9	
N3	29	1604.2	671.7		1510.2	620.6		1944.3	888.0		454.8	270.0	
Bladder cancer													
T stage				0.7482**			0.7320**			0.5790**			0.1793**
Tis	2	1736.6	429.9		1568.4	406.3		2046.9	355.0		395.2	119.1	
T1	4	1326.0	525.4		1306.3	517.3		1717.4	525.6		299.2	222.9	
T2	10	1833.5	832.3		1710.5	759.0		2207.7	1074.9		451.0	160.6	
T3	7	1812.3	522.3		1833.4	506.3		2541.3	630.6		444.4	141.7	
T4	8	1800.7	582.9		1630.9	544.2		2098.4	638.9		596.2	263.3	
N stage				0.8224**			0.7579**			0.7786**			0.1010**
N0	10	1665.1	479.6		1535.2	446.2		2158.4	619.1		436.1	168.2	
N1	5	1602.0	482.3		1549.8	507.6		1872.4	726.4		411.2	173.2	
N2	8	1813.4	964.3		1806.0	881.1		2333.5	1107.6		400.4	180.1	
N3	5	1937.5	428.0		1761.7	394.4		2135.4	234.1		671.9	287.7	
Pancreatic cancer													
T stage				0.7748**			0.8479**			0.8488**			0.8453**
T1	1	1398.3			1294.2			1400.7			419.7		
T2	12	1823.2	708.6		1730.9	647.8		2413.7	897.1		476.3	139.5	
T3	8	2031.6	1477.7		1891.1	1421.5		2693.9	2009.0		556.0	286.2	
T4	15	2279.0	1509.8		2077.8	1423.9		2741.7	1919.3		595.7	506.3	
N stage				0.1928*			0.1448*			0.1243*			0.0671*
N0	9	2663.9	1855.8		2564.3	1749.6		3475.3	2290.2		699.2	377.1	
N1	19	1756.4	837.8		1598.2	738.6		2133.6	964.7		428.6	152.1	

* Student *t*-test.

** One-way ANOVA.

### Impact of other factors on PLA_2_ activity

**Figure 3:**
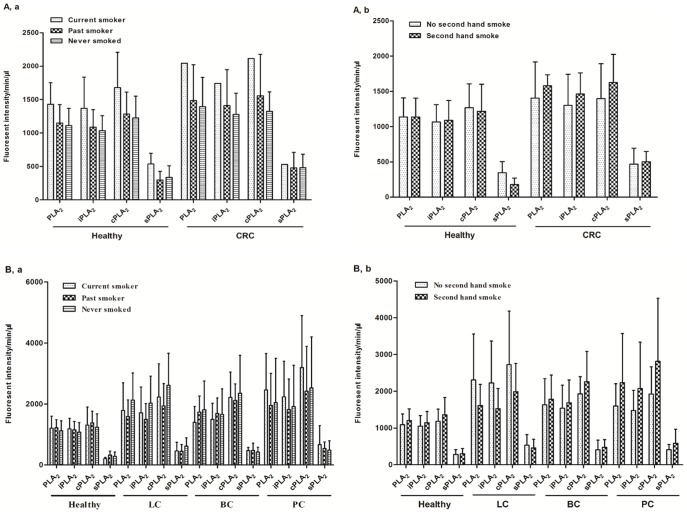
The influence of smoking on plasma PLA_2_ activities. PLA2 activities in healthy and CRC samples with different smoking statuses(a)and second hand smoking statuses(b). A. Comparison of PLA2 activities between different smoking statuses(a) and second hand smoking statuses(b) in participants with LC, BC, and PC.

We have collected other factors, which might influence PLA_2_ activities, including smoking, alcohol consumption, and body-mass index (BMI). For the CRC study, from the 80 subjects with available smoking information (27 CRC and 53 healthy cases), the majority (96%) of people did not smoke when the samples were collected, with an average 50.5% people never smoked and 45.7% of past smoker, and only 9.4% and 19.2% people had second hand smoking exposure in each group. The smoking statuses were similarly distributed in the two groups (*P* values >0.05; detailed data in **Table 4**) and there were no statistical differences in PLA_2_ activities among different smoking statuses (**Fig. 3A**). In the second set of study, the smoking statuses were significantly different between healthy controls and each of the cancer groups with higher percentages of people who were current, past, or second-hand smokers in cancer groups (*P* values <0.05, except in one comparison, **Table 5**). Nevertheless, no statistical differences were observed in the PLA_2_ activities among different smoking statuses, supporting the concept obtained from the first set of study that plasma PLA_2_ activities are not affected by smoking (**Fig. 3B**).

**Table 4:** Smoking, alcohol consumption, and the BMI status of participants from IUSM*.

	Healthy		CRC		
	No.	%	No.	%	*P* value
Smoking status					
Current smoker	2	3.8	1	3.7	0.4609
Past smoker	18	34.0	13	48.2	
Never smoked	33	62.3	13	48.2	
Second hand smoke					0.2185
No	48	90.6	21	80.8	
Yes	5	9.4	5	19.2	
Alcohol					0.2542
No	6	11.3	1	3.7	
Yes	47	88.4	26	96.3	
BMI (Mean ± SD)	28.2±6.96		25.4±4.01		0.0272

* These data are available from a subset of the participants.

**Table 5:** Smoking and alcohol consumption status of participants from HOG study.

	Healthy		LC			BC			PC		
	No.	%	No.	%	*P* value	No.	%	*P* value	No.	%	*P* value
Smoking status					<0.0001			0.0017			0.016
Current smoker	4	5.1	29	30.5		4	12.9		7	18.4	
Past smoker	25	31.6	58	61.1		19	61.3		16	42.1	
Never smoked	50	63.3	8	8.4		8	25.8		15	39.5	
Second hand smoke					<0.0001			0.0091			0.0741
No	32	40.5	10	10.5		4	13.8		9	23.7	
Yes	47	59.5	85	89.5		25	86.2		29	76.3	
Alcohol					<0.0001			0.0018			<0.0001
No	23	29.6	67	70.5		19	61.3		31	81.6	
Yes	56	70.4	28	29.5		12	38.7		7	18.4	
BMI (Mean ± SD)	28.3±5.55		26.5±6.76		0.0707	28.3±5.01		0.9625	25.9±6.17		0.0374

LC: Lung cancer; BC: Bladder cancer; PC: Pancreatic cancer.

All *P* values are comparisons between the cancer group and the healthy group.

**Figure 4:**
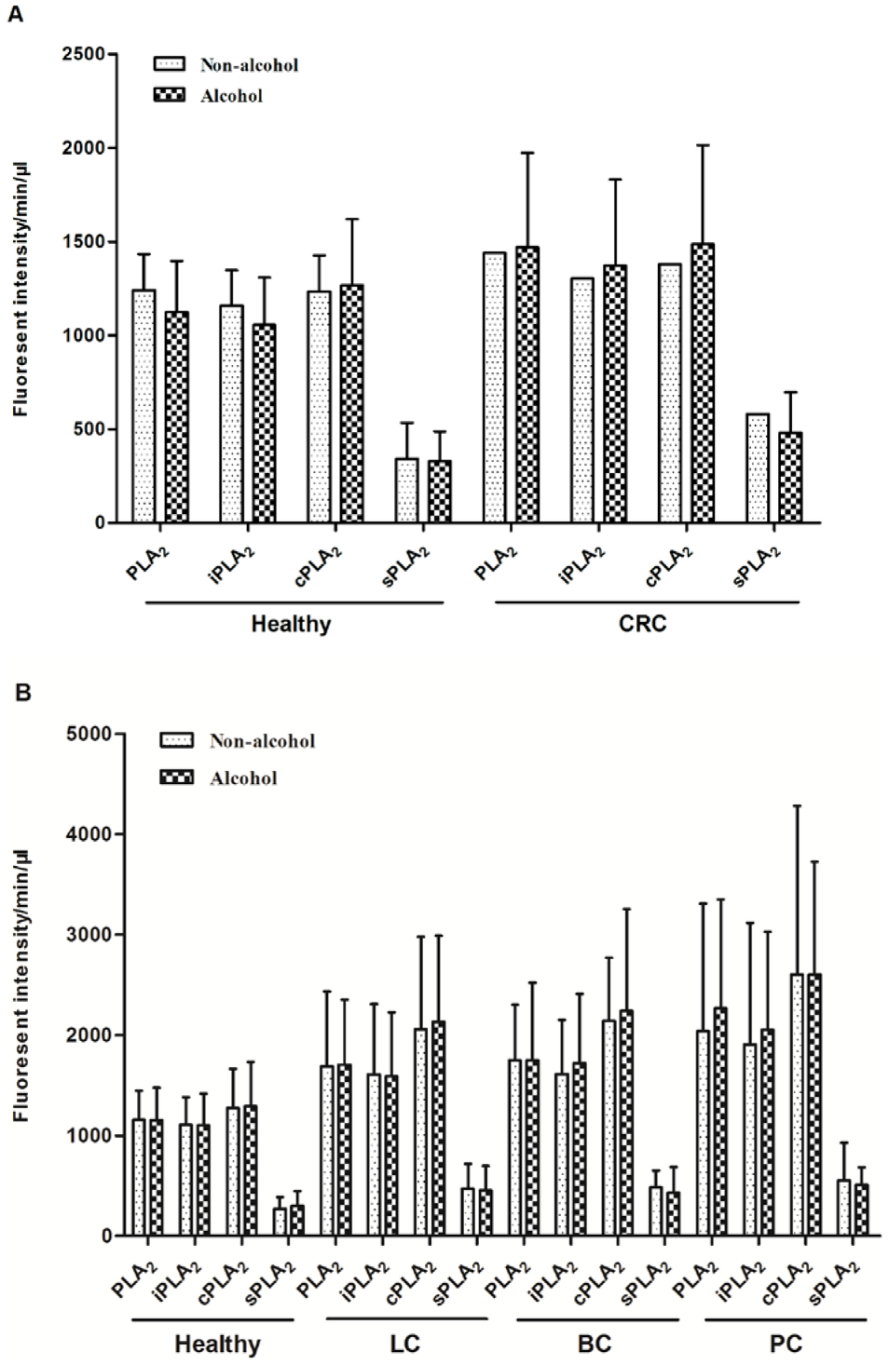
The influence alcohol consumption on plasma PLA_2_ activities. Comparison of PLA2 activities between “non-alcohol” and “alcohol” groups with the healthy and CRC participants. A. Comparison of PLA2 activities between “non-alcohol” and “alcohol” groupsin the second set of studies with healthy, LC, BC, and PC participants.

**Figure 5:**
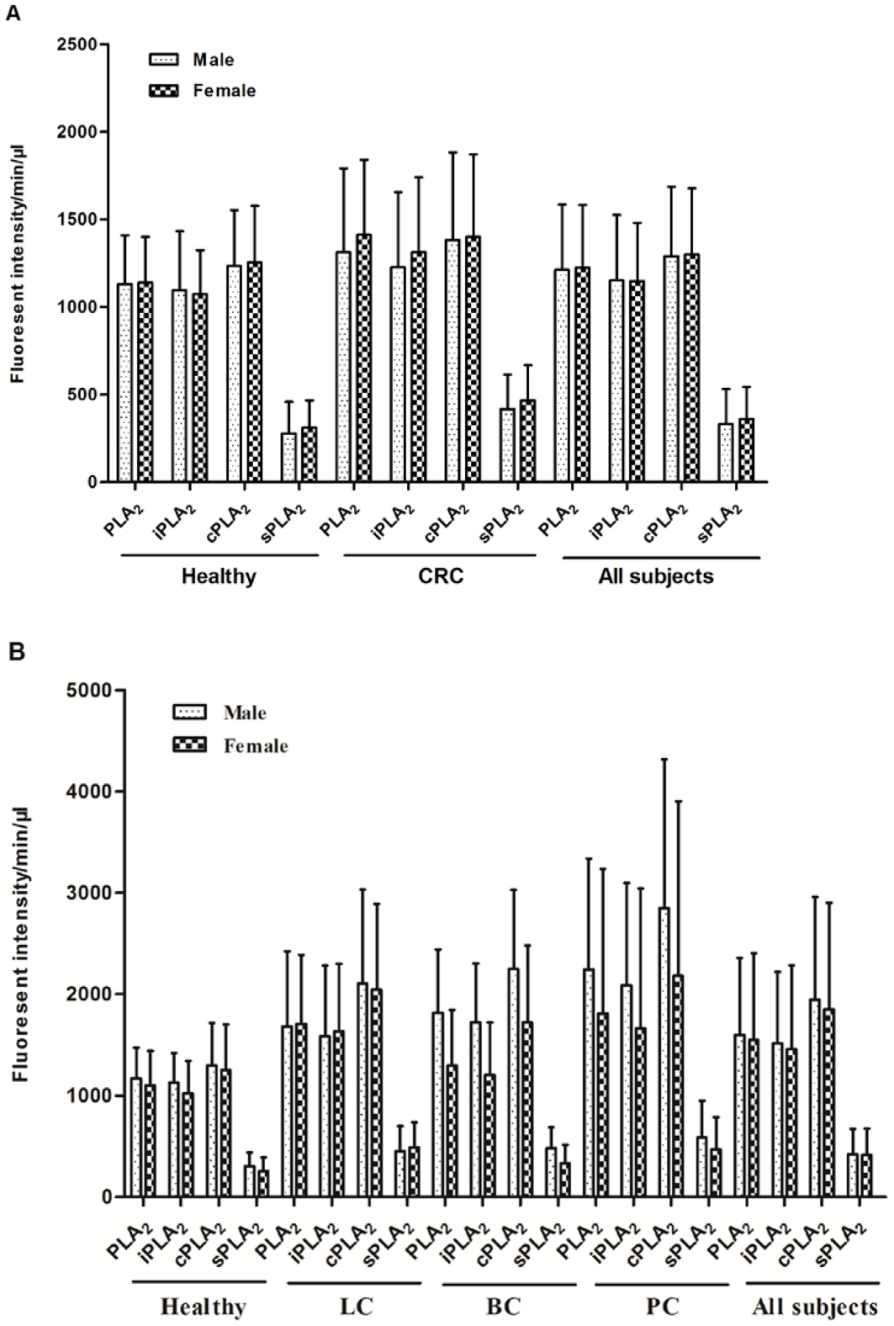
PLA_2_activities were not significantly different between male and female. PLA2 activity comparison in males and females in the healthy and the CRC groups. A. PLA2 activity comparison in males and females in the healthy and other cancer groups. Data from all subjects in each set of studies are also presented.

Alcohol consumption in the control and CRC groups were not significantly different (**Table 4**). The alcohol consumers in the CRC groups tend to have higher levels of plasma PLA_2_ activities, but the differences were not statistically significant (**Fig. 4**). However, due to the low numbers in non-alcohol consumption group (n = 7), we should interpret these data with caution. For other cancers, similar results were obtained. Although there were significant higher percentages of alcohol consumption subjects in other cancer groups (**Table 5**), the PLA_2_ activities were not significantly related to alcohol (**Fig. 5**).

**Figure 6:**
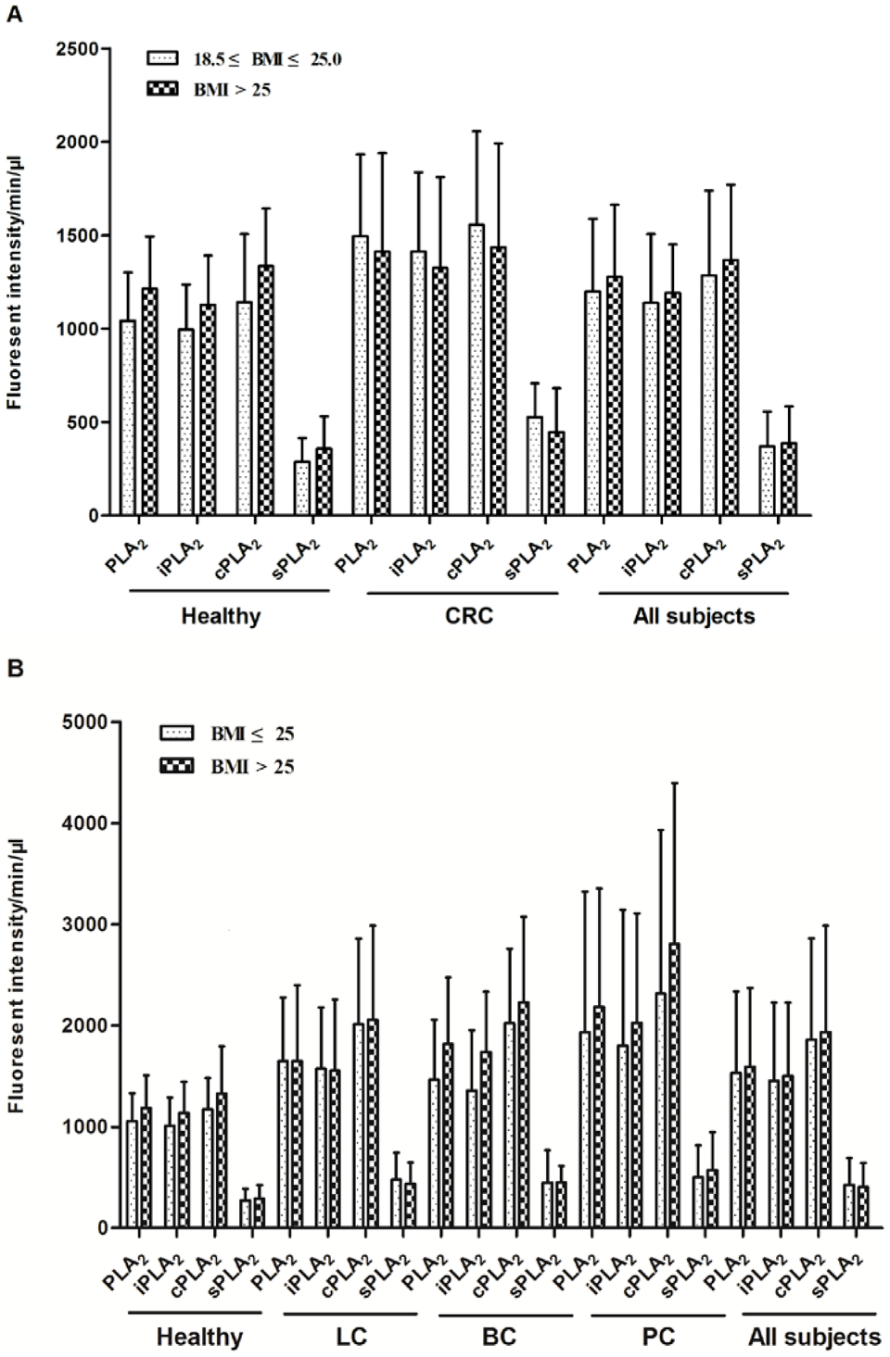
Comparison of PLA_2_ activities among different BMI groups. The subjected in healthy and CRC groups were divided into two groups: BMI 18.5 to <25 (For all subjects, n = 43) and BMI>25 (For all subjects, n = 80). A. The subjected in healthy and other cancer groups were divided into two groups: BMI≤25 (For all subjects, n = 80) and BMI>25 (For all subjects, n = 148).

The differences in PLA_2_ activities between sexes did not reach a statistical significance in the control or any cancer groups in either set of studies (**Figs. 5A** and **5B**). Similarly, there was no difference or correlation in PLA_2_ activities in subjects with BMI <25 vs. BMI ≥25 groups in the CRC study (**Figs. 6A** and **6B**), although the mean BMI in the CRC and PC groups were significantly less than those in control groups (**Tables 4** **and** **5**).

**Figure 7:**
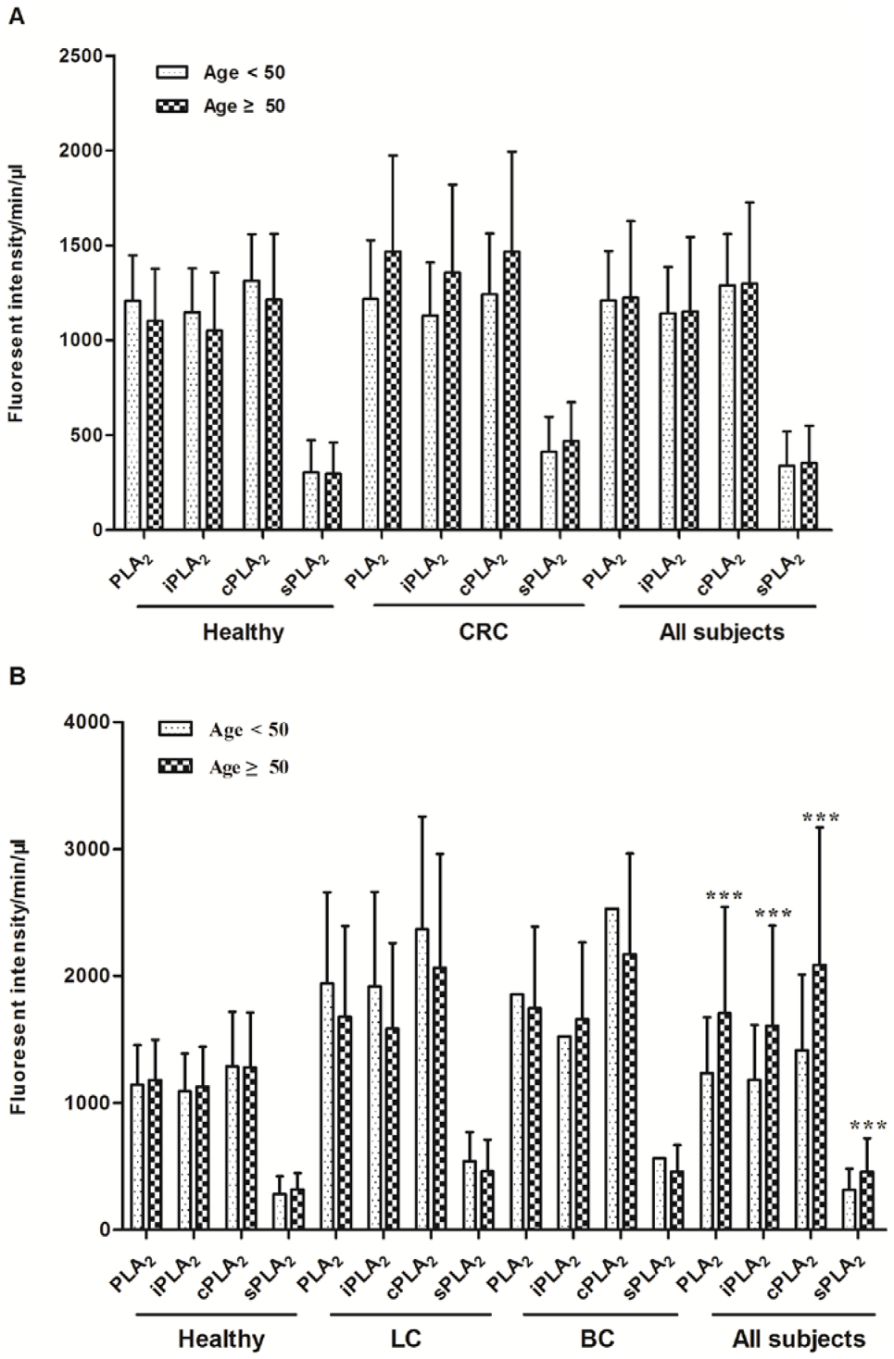
PLA_2_activities were not significantly correlated to ages. The participants in healthy and CRC groups were divided into two groups: age<50 (For all subjects, n = 42) and age ≥50 (For all subjects, n = 153). Student t-test was performed to analyze the differences between these two groups. A. The participants in healthy and other cancer groups were divided into two groups: age<50 (For all subjects, n = 61) and age ≥50 (For all subjects, n = 144). Student t-test was performed to analyze the differences between these two groups. ***P<0.001.

Age is one of the most clearly identified risk factors for developing CRC and other cancers. The American Cancer Society and the U.S. Preventive Services Task Force recommend that people receive colonoscopy screenings every 10 years beginning at age 50. We thus analyzed the age effects on PLA_2_ activities by dividing the subjects into two groups (age <50 years, n = 37 and ≥50 years, n = 78). Although there was a trend of increased PLA_2_ activities in the ≥50 years-old group in the CRC group, the differences were not statistically significant (**Fig. 7A**). Similarly, no difference was detected between the two age groups in the LC, BC, and PC set, although when all the subjects (including healthy controls) were combined, there were significant increases in PLA_2_ activates in the older subjects (**Fig. 7B**).

A direct comparison of the two sets of healthy controls showed that although their sex, age, second-hand smoking, and alcohol consumption were different, there were no differences in any PLA_2_ activity measured (**Table 6**). This further supports that plasma PLA_2_ activities are not significantly affected by age, sex, alcohol consumption, and/or smoking.

**Table 6:** Comparison of two independent sets of healthy controls.

	1^st^ set		2^nd^ set		
	No.	%	No.	%	*P* value
Gender					<0.0001
Male	31	40.3	61	77.2	
Female	46	59.7	18	22.8	
Smoking status					
Current smoker	2	3.8	4	5.1	0.889
Past smoker	19	35.8	25	31.6	
Never smoked	32	60.4	50	63.3	
Second hand smoke					<0.0001
No	48	90.6	32	40.5	
Yes	5	9.4	47	59.5	
Alcohol					<0.0001
No	6	11.3	23	29.6	
Yes	47	88.4	56	70.4	
Age (Mean ± SD)	51.6±13.5		42.7±12.03		<0.0001
PLA_2_ activities(Mean ± SD)					
PLA_2_	1135.9±266.2		1156.3±311.8		0.660
iPLA_2_	1082.0±286.1		1105.4±301.6		0.620
cPLA_2_	1246.1±318.7		1287.8±425.9		0.488
sPLA_2_	299.1±164.7		293.9±137.3		0.828

### The classification performance of plasma PLA_2_ activities

**Figure 8:**
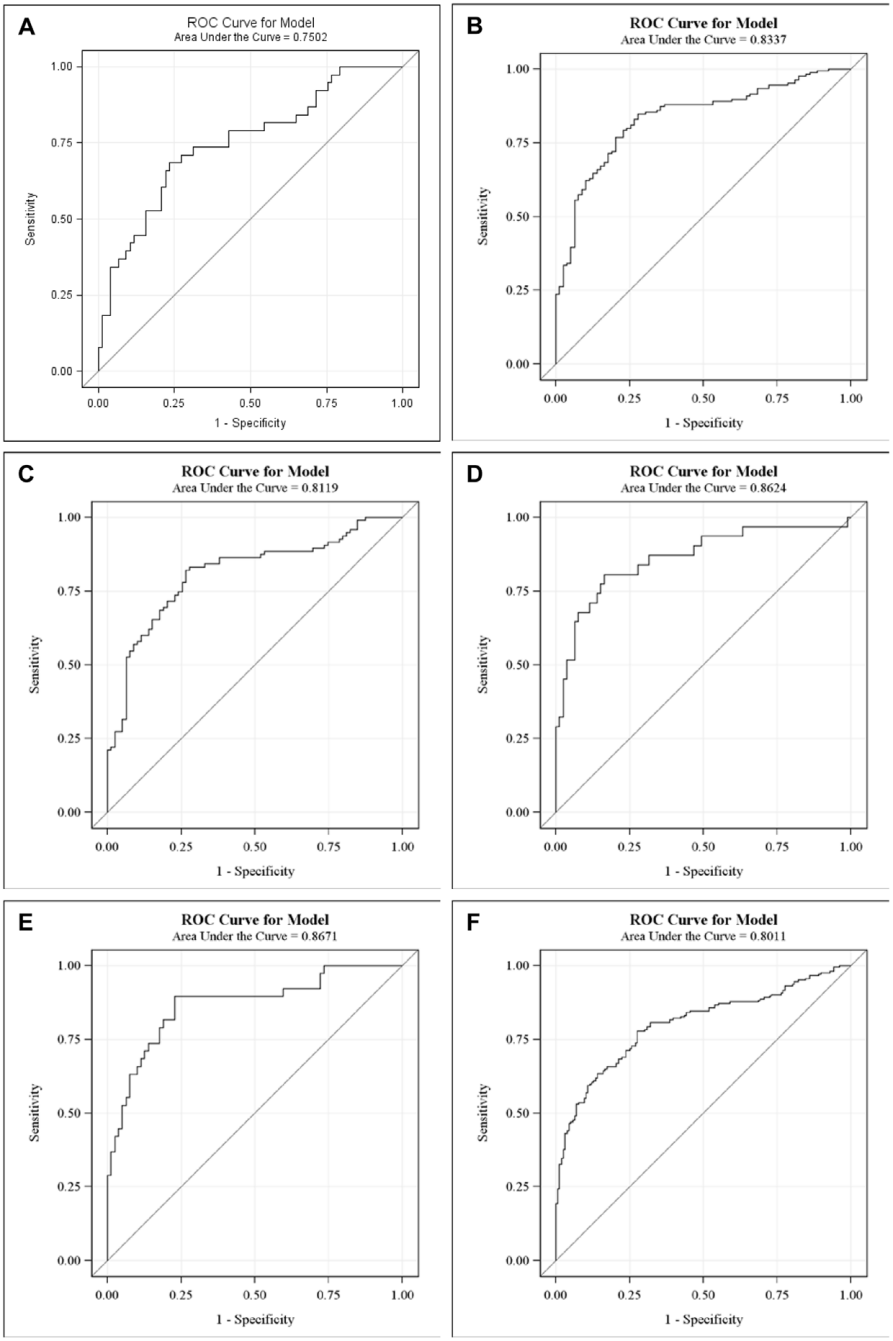
ROC curves of plasma PLA_2_ activities in different groups. CRC cases vs. healthy subjects. A. All cancers vs. healthy subjects in the 2nd set. B. Lung cancer vs. healthy subjects. C. Bladder cancer vs. healthy subjects. D. Pancreatic cancer vs. healthy subjects. E. All cancers vs. all healthy subjects with combined two sets.

Logistic regression was used to evaluate the classification performance of PLA_2_ activities in cancers. All four PLA_2_ measurements were kept as predictors regardless of their significance. The classification performances were summarized by receiver operating characteristic (ROC) curves (**Fig. 8**). Prediction formulas were generated using the parameter estimates.

The prediction formula to separate CRC cases from healthy subjects is:
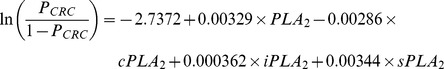
where “*P*_CRC_” stands for the probability of having CRC. The ROC curve shown in **Fig. 8A** has an area under the curve (AUC) = 0.7502.

Since external validations would require an independent study, we instead adopted internal methods to validate the classification performances. Among different internal validation methods, it has been shown that bootstrap outperforms jackknife, cross-validation and data-splitting methods [19]. Therefore, bootstrap was adopted for validation. In particular, the prediction formula was obtained by fitting the same model for each bootstrapped sample. The formula was applied to the original data to calculate the prediction probabilities. The performance for this particular bootstrap sample was summarized by AUC of the ROC curve. The overall performance was evaluated by summarizing AUCs across 1,000 bootstrapped samples. In 95% of the 1000 bootstrapped samples, AUCs are higher than 0.7143 for CRC vs. healthy. Therefore, these results internally confirm the classification performances of the models developed above.

The prediction formula and the ROC curve to separate all cancer cases from healthy subjects in the second set of study are as follows and shown in **Fig. 8B**, with an AUC = 0.8337.
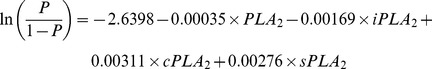
This performance was validated in 1000 bootstrapped samples. In 95% of the 1000 bootstrapped samples, AUCs are higher than 0.8205 for LC, PC, BC combined vs. the second set of healthy controls.

The prediction formulas and the ROC curves for the separate LC, BC, and PC cases from healthy subjects in the second set of study are listed below and **Figs. 8C** to **8E**, with AUCs >0.811, indicating good classification performances of the tests. For LC versus healthy control, the AUC = 0.8119 (**Fig. 8C**). In 95% of the 1000 bootstrapped sample, AUCs are higher than 0.7923.
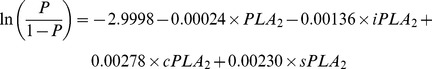
For BC versus healthy control, the AUC = 0.8624 (**Fig. 8D**). In 95% of the 1000 bootstrapped sample, AUCs are higher than 0.8161.
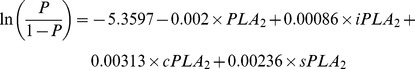
For PC versus healthy control, the AUC = 0.8671. In 95% of the 1000 bootstrapped sample, AUCs are higher than 0.8426.
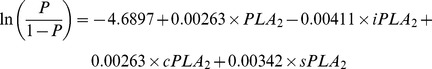



Finally, since we have found that the PLA_2_ activities were essentially no difference in the two sets of healthy controls, we combined all healthy controls and all cancer cases in both sets of studies and generated a combined formula, with an AUC = 0.8011 (**Fig. 8F**). In 95% of the 1000 bootstrapped samples, AUCs are higher than 0.7907.
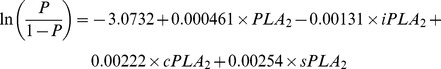
The sensitivities and specificities can be obtained from the ROC curves shown in **Fig. 8**. For the CRC set of study, 60.5% sensitivity and 77.9% of specificity were obtained (**Table 7**). For the 2^nd^ set of study and the data combined for both sets, sensitivities >80% and the specificities >66% were obtained for all cases (**Table 7**).

**Table 7:** Sensitivities and specificities of PLA_2_ to distinguish different cancers from healthy cases.

	Without other parameters		With other parameters	
	Sensitivity	Specificity	Sensitivity	Specificity
First set				
CRC vs. Healthy	60.5%	77.9%	63.0%	75.5%
Second set				
All cancer vs. healthy	81.1%	72.2%	87.2%	88.6%
LC vs. healthy	82.1%	67.1%	85.3%	91.1%
BC vs. healthy	80.6%	75.9%	87.1%	87.3%
PC vs. healthy	81.6%	77.2%	86.8%	96.2%
Combined two sets				
Cancer vs. healthy	80.2%	66.7%	83.2%	81.1%

### The potential contributions of other factors

Although most other demographic and/or environmental factors tested were not significantly different in cancer and control groups (**Figs 3** **–** **7**), we tested their potential contributions to the performance when they are evaluated at individual levels. We kept all four PLA_2_ measurements in the models, but let other predictors subject to model selections at the significant level 0.05.

**Figure 9:**
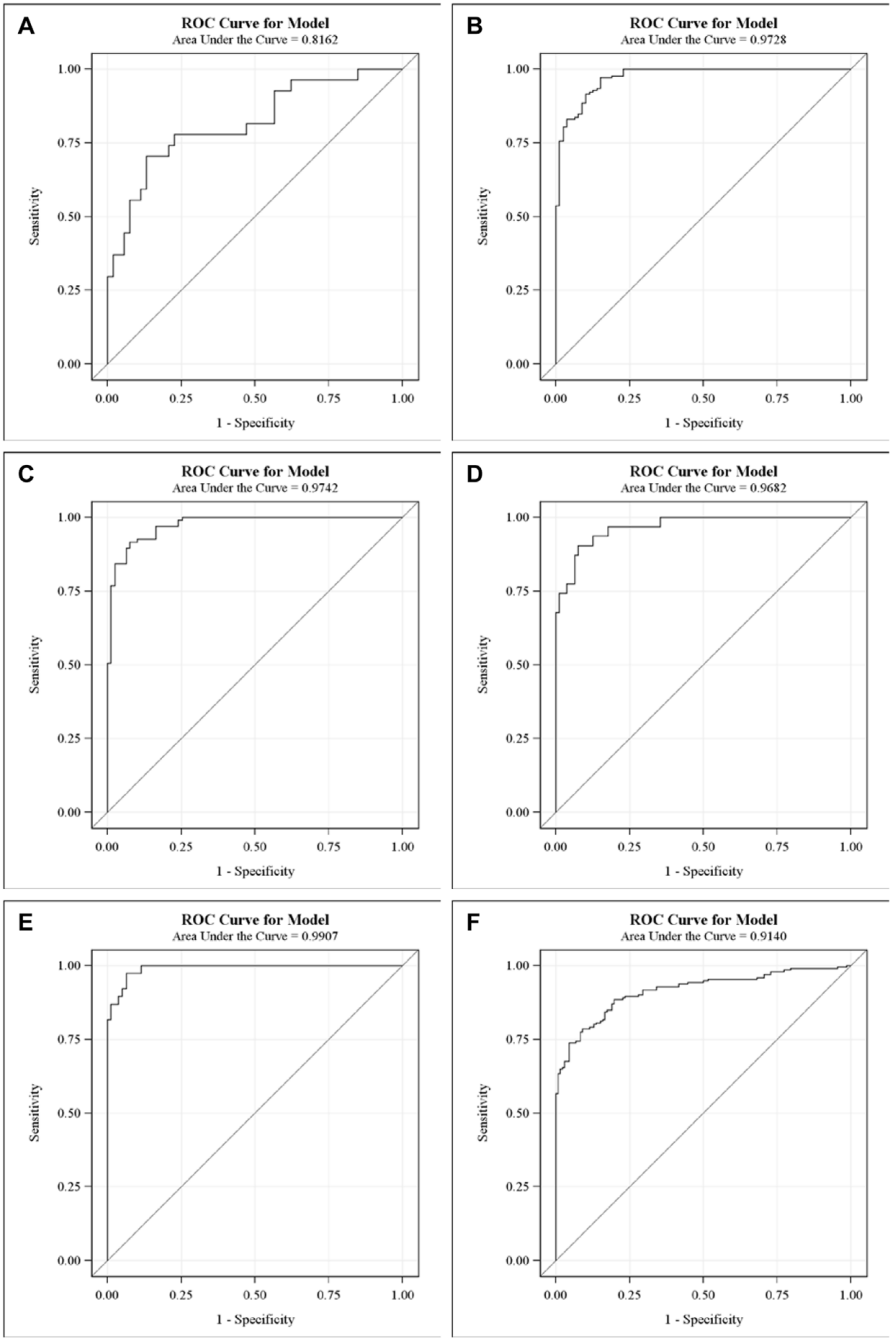
ROC curves of plasma PLA_2_ activities with additional parameters. CRC cases vs. healthy subjects. A. All cancer cases vs. healthy subjects in the second set. B. Lung cancer vs. healthy subjects. C. Bladder cancer vs. healthy subjects. D. Pancreatic cancer vs. healthy subjects. E. All cancers vs. all healthy subjects with combined two sets.

The prediction formula and the ROC curve to separate CRC from healthy subjects in the first set of study are as follows and shown in **Fig. 9A**, with an AUC = 0.8162. In 95% of the 1000 bootstrapped sample, AUCs are higher than 0.7589.
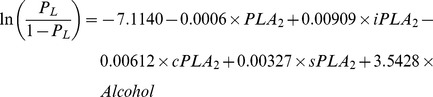
The prediction formula and the ROC curve to separate all cancer cases from healthy subjects in the second set of study are as follows and shown in **Fig. 9B**, with an AUC = 0.9728.
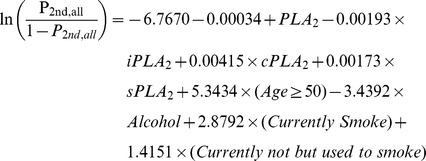
This performance was validated in 1000 bootstrapped samples. In 95% of the 1000 bootstrapped samples, AUCs are higher than 0.9625 for LC, PC, and BC combined vs. the second set of healthy controls.

The prediction formulas and the ROC curves for the separate LC, BC, and PC cases from healthy subjects in the second set of study are listed below and **Figs. 9C** to **9E**, with AUCs >0.968, indicating high sensitivities and specifies of the tests. For LC versus healthy control, the AUC = 0.9742. In 95% of the 1000 bootstrapped sample, AUCs are higher than 0.9598.
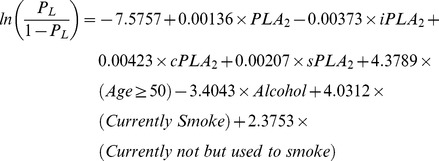
For BC versus healthy control, the AUC = 0.9682. In 95% of the 1000 bootstrapped sample, AUCs are higher than 0.9398.
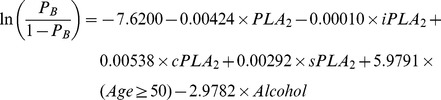
For PC versus healthy control, the AUC = 0.9907. In 95% of the 1000 bootstrapped sample, AUCs are higher than 0.9740.
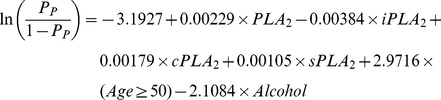



Finally, since we found that the PLA_2_ activities were essentially no difference in the two sets of healthy controls, we combined all healthy controls and all cancer cases in both sets of studies and generated a combined formula, with an AUC = 0.9140 (**Fig. 9F**). In 95% of the 1000 bootstrapped samples, AUCs are higher than 0.9018.
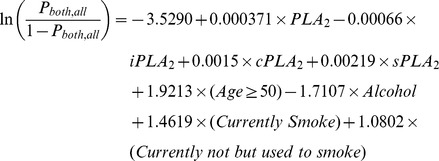
The sensitivities and specificities obtained with these ROC curves with additional parameters are increased to >85% (except CRC vs. healthy cases; **Table 7**).

## Discussion

In this work, we have presented the first measurements of plasma natural and subtype PLA_2_ activities and their performances as potential markers for four different cancers, CRC, LC, BC, and PC. We have measured the PLA_2_ activities in more than 20 blood samples with or without adding 1.2 mM calcium (the natural ionized calcium concentration in blood) to the final assay mixtures and did not detect any significant differences. Hence, we define the PLA_2_ activities obtained without any additives as the “natural” PLA_2_ activity. We have selected optimal conditions to measure each sub-family of PLA_2_ activities. These modified conditions do not co-exist in the blood. Therefore, it is not surprising that the sum of the subfamily PLA_2_ activities is greater than the “natural” PLA_2_ activity. However, it is important to note that although modified conditions were used to obtain the activities for subfamilies of PLA_2_s, the final results are from the natural PLA_2_s present in the blood samples.

While the advantages of using convenient serologic markers are obvious, no reliable blood markers for any of these cancers are currently available. For CRC, developing/validating non-invasive or minimally invasive detection methods is a major focus in the field. Current methods include stool and blood tests, such as fecal immunochemical tests (FITs) and guaiac-based fecal occult blood testing (got). Although the specificity is high (>85%), these tests have a low sensitivity (<23%) for colorectal adenomas and are thus unlikely to be able to increase the early detection of CRC [20], [21]. Genetic stool tests detect mutations in stool that can be found in CRC. A 21 genetic change test was found to be superior to guaiac fecal occult blood test (gFOBT) for the detection of CRC [20]. However, the sensitivity only reached 51.6% [20]. The plasma PLA_2_ activities tests presented show promising initial results in separating healthy controls from cancer patients with sensitivities and specificities approximately 70%. The majority solid cancers are highly heterogeneous, which is one of the major reasons that any single or small set of markers can hardly detect any specific cancer with very high sensitivity and specificity. Testing the potential complementary values of other identified markers in CRC and other cancers will be of high significance. Interestingly, our results suggest that PLA_2_ activities are independent of several common demographic and environmental factors. In addition, we have compared 11 CRC blood samples collected before and after surgeries and found that 7 of 11 had reduced PLA_2_ activities (26.1±10.9% reduction) and 4 of 11 had increased PLA_2_ activities (37.7%±11.7%). Among them, disease progression data was available for only 6 subjects (3 from each group). Interestingly, all 3 subjects from the reduced PLA_2_ activity group had chemotherapy and complete response and all 3 subjects from the increased PLA_2_ activity group had chemotherapy and disease progression. These data were from a very limited number of subjects, but suggest that the prognostic value of PLA_2_ activities warrants further testing. We are fully aware that our studies are limited to the cohort size and external validation in independent and larger scale studies will be critical and hope that our report will promote such studies.

Is a marker which may detect multiple types of cancers useful? The answer is likely to be yes. Even after decades of efforts, the success rate in finding highly specific markers for specific cancers has been low. Prostate-specific antigen is an exception, but even the value of this marker as a screening tool in prostate cancer has been recently questioned [22]. Many tumor markers are known to affect multiple cancers. Sensitive, minimally invasive, reproducible, and cost-effective blood biomarkers to detect multiple malignancies are likely to be clinically significant and highly valuable as routine first line detection. In addition, the PLA_2_ activity test is very easy to perform in any laboratory with a fluorescent plate reader, and would be feasible to develop into an automated test. Moreover, very small amounts samples (1–10 µL of plasma) are needed to perform the test and the results can be obtained in 1–2 hrs. Additional markers or more specific modalities, such as colonoscopy or imaging are likely to be needed to detect specific cancers.

Another major advantage of the test is that it is reproducible, robotic, and stable. It is well-recognized that many blood markers are sensitive to how blood samples are handled, processed, and stored. It is almost impossible to unify these procedures in the USA or anywhere in the world. Freeze-and-thaw is another well-known factor affecting marker stability. These reasons account, at least in part, for the fact that although thousands of markers have been reported in the past decades, seldom have any of them been cross-validated in different centers and moved to the clinic. We have used two completely independent sets of human plasma samples, which were processed somewhat differently (see Method for details) and show that the PLA_2_ activities are highly compatible, consistent in healthy controls, and are independent of several demographic and environmental factors, making them more likely to be useful markers. More independent studies with large samples sizes need to be conducted to further evaluate the clinical significance of our finding reported here.

A potential caveat of the clinical usage of PLA_2_ activity is that these enzymes are known to be involved in inflammation and may be elevated in patients with benign inflammatory diseases. This needs to be experimentally tested in clinical samples. However, there are mounting lines of evidence supporting the strong causal connections between inflammation and cancers [23]. In particular, chronic inflammation plays a pivotal role in the development of CRC in patients with inflammatory bowel disease [24]. The connection between inflammation and lung cancer has been shown not coincidental but may indeed be causal [23]. In addition, non-steroidal anti-inflammatory drugs has been associated with reduced risk to developing many types of cancers [25]. Thus, future studies on the significance of elevated PLA_2_ activity in disease detection and progression prediction will be highly interesting.

Our results also strongly imply that the PLA_2_ activities (which may or may not correlate to their RNA or protein expression levels) are potential targets for cancer treatment tested here. Most, if not all previous PLA_2_ assays conducted in tissues or cell lines mainly focus on their expression levels using PCR and IHC, which are gene/protein specific, time-consuming, costly, and require relatively large amount of samples to cover different isoforms of PLA_2_s. Since there are more than 30 PLA_2_s, none of the previous studies provide an overall picture of PLA_2_s in any cancer. More importantly, their enzymatic activities, but not necessarily their RNA and/or protein expression levels, are directly related to the biological effects, since PLA_2_ activities are well-known to be regulated post-transcriptionally [26], [27]. This concept has been supported in our recent pre-clinical (mouse models) and human sample studies in ovarian cancer [16] and remain to be tested further in other cancers.
